# Non-excisional laser therapies for hemorrhoidal disease: a systematic review of the literature

**DOI:** 10.1007/s10103-020-03142-8

**Published:** 2020-09-10

**Authors:** Gregoire Longchamp, Emilie Liot, Jeremy Meyer, Christian Toso, Nicolas C. Buchs, Frederic Ris

**Affiliations:** Division of Digestive Surgery, Geneva University Hospitals and Medical School, Geneva, Switzerland

**Keywords:** Hemorrhoids, Laser, Complications, Outcomes

## Abstract

Non-excisional laser therapies are emerging treatment for grades II and III hemorrhoidal disease (HD). However, so far, their efficiency is based on low-level evidence. Therefore, we aimed to systematically review the efficiency of non-excisional laser therapies for HD. MEDLINE/Pubmed, Web of science, Embase, and Cochrane were searched from database implementation until the April 17th, 2020. We included studies reporting at least one of surgical indicators of postoperative outcomes of laser therapies, encompassing laser hemorrhoidoplasty (LH) and hemorrhoidal laser procedure (HeLP). Fourteen studies describing LH and HeLP were included, representing 1570 patients. The main intraoperative complication was bleeding (0–1.9% of pooled patients for LH, 5.5–16.7% of pooled patients for HeLP). Postoperative complications occurred in up to 64% of patients after LH and 23.3% after HeLP. Resolution of symptoms ranged between 70 and 100% after LH and between 83.6 and 90% after HeLP. Moreover, four randomized controlled trials included in our review reported similar resolution after LH compared with hemorrhoidectomy or mucopexy and after HeLP compared with rubber band ligation. Recurrence rate was reported to range between 0 and 11.3% after LH and between 5 and 9.4% after HeLP. When compared with hemorrhoidectomy, LH showed conflicting results with one randomized controlled trial reporting similar recurrence rate, but another reporting decreased recurrences associated with hemorrhoidectomy. Laser therapies showed lower postoperative pain than hemorrhoidectomy or rubber band ligation. LH and HeLP are safe and effective techniques for the treatment of grades II and III HD.

**Electronic supplementary material:**

The online version of this article (10.1007/s10103-020-03142-8) contains supplementary material, which is available to authorized users.

## Introduction

Hemorrhoidal disease (HD) is frequent, with an estimated prevalence of 4.4% among the US population [1]. HD is the consequence of an increased inflow into the superior rectal artery, which causes dilatation of the hemorrhoidal plexus. Moreover, degradation of the supportive tissue results in sliding down of hemorrhoids [2]. Hemorrhoids are classified as grade I when they are seen during anoscopy as congested veins, grade II when they prolapse but spontaneously reduce, grade III when they prolapse and need manual reduction, and grade IV when they are irreducible [3]. HD, defined as symptomatic hemorrhoids, can present with pain, itching, bleeding, discharge, or prolapse [4].

Initial treatment of HD consists of lifestyle modifications and administration of phlebotonics. After failure of conservative management, HD is treated with interventional therapies [4]. Open hemorrhoidectomy (HC) was first described in 1937 by Milligan-Morgan [5] and is still considered as the gold standard interventional therapy for advanced stages of HD. However, significant postoperative pain and complications were associated with excision of hemorrhoidal tissue. Therefore, various non-excisional therapies have been developed, such as rubber band ligation (RBL), mucopexy (MP), and more recently laser therapies [4, 6].

Non-excisional laser therapy was initially described in 1998 by Barr et al. [7] with an experimental animal study. Administration of a pulsed laser energy to the submucosal pig rectal tissue allowed coagulation of vessels, with limited damage to the surrounding tissue. Latter, non-excisional laser therapy was applied in humans, with laser hemorrhoidoplasty (LH) first described in 2007 by Karahaliloglu et al. [8]. During LH, a laser fiber is introduced through a skin incision at the hemorrhoidal base, and hemorrhoidal cushions are coagulated. Hemorrhoidal laser procedure (HeLP) constitutes another non-excisional laser therapy for the treatment of HD, first described in 2009 by Salfi et al. [9]. During this procedure, a Doppler identifies the terminal branches of the superior rectal artery, which are coagulated with a pulsed laser energy. Both techniques allow obliteration and retraction of the hemorrhoidal plexus. Moreover, they were shown to be safe and effective for the treatment of HD [8, 9].

Therefore, non-excisional laser therapies constitute interventional therapies for the treatment of HD. However, their recommendation is based on low level of evidence [4, 6]. For the purpose of strengthening the evidence for the benefits of laser therapies, we aimed to systematically review the outcomes of LH and HeLP for the treatment of HD. According to population, intervention, comparison, outcome (PICO) framework, our question was: in patients with HD undergoing non-excisional laser therapies, what are the postoperative outcomes?

## Materials and methods

This systematic review adheres to the recommendations of the Preferred Reporting Items for Systematic Review and Meta-analyses (PRISMA) statement [10] (Supplementary Table 1).

### Literature search and study selection

Human studies written in English published before April 17th, 2020 were looked for in MEDLINE/Pubmed, Web of science, Embase, and Cochrane. The search strategy was designed and independently conducted by two authors (GL, EL). The following medical search headings and keywords were used: “hemorrhoids” in MeSH terms; and “haemorrhoid*” OR “hemorrhoid*” OR “hemorrhoidal disease” OR “haemorrhoidal disease” AND “laser” OR “laser hemorrhoidoplasty” OR “hemorrhoidal laser procedure” in non-MeSH terms. The reference lists of included articles were further screened for additional eligible publications.

### Outcomes of interest

The aim of the study was to systematically review the outcomes of laser therapies for the treatment of HD, including LH and HeLP.

Primary endpoints were the surgical indicators of postoperative outcomes, including

- Improvement, defined as postoperative decrease of HD symptoms or grade adapted from the Goligher classification [3];
- Persistence, defined as postoperative presence of symptoms or prolapse;
- Resolution, defined as postoperative absence of symptoms or prolapse;
- Recurrence, defined as reappearance of HD, after a resolution;
- Reoperation, defined as any procedure performed for HD after the laser therapy.

Secondary endpoints included

- Perioperative characteristics;
- Postoperative pain and return to normal activities;
- Intraoperative and postoperative complications, defined as any deviation from the normal postoperative course. Therefore pain, tenesmus, and dyschezia that resolved spontaneously without treatment were not considered as complications.

### Inclusion criteria

Original publications were eligible only if they fulfilled the following criteria: (i) they reported outcomes of laser therapy for HD and (ii) they reported at least one of the primary endpoint. Articles were included regardless of the design and the size of the study population.

### Exclusion criteria

The exclusion criteria were as follows: studies reporting (i) laser hemorrhoidectomy or infrared therapy; (ii) laser therapy performed with an associated procedure or for another anorectal pathology than HD; and (iii) conference abstracts, protocols, and editorials.

### Data extraction

Two reviewers (GL, EL) extracted the following data: general and methodological information of the study, baseline characteristics of the study population, surgical indicators of postoperative outcomes, perioperative characteristics, complications, postoperative pain, and return to normal actives. Details of extracted data are reported in the Supplementary Table 2.

## Results

### Literature search and study characteristics

**Fig. 1:**
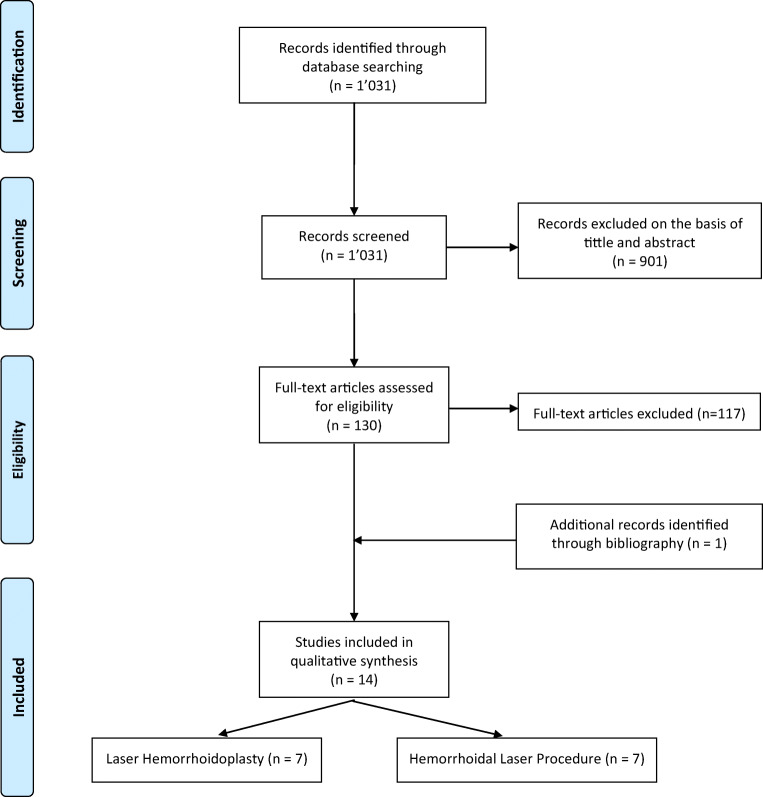
Preferred reporting items for systematic review and meta-analyses (PRISMA) flowchart showing selection of publications for review

The initial search identified 1031 studies. After title and abstract review, 901 studies were excluded. The remaining 130 studies were fully reviewed. Of these, 117 were excluded because they reported laser hemorrhoidectomy (42 studies), infrared therapy (68 studies), associated procedure (three studies), or laser therapy for other pathology than HD (four studies). Reviewing of references identified one study [9]. Finally, 14 studies [8, 9, 11–22] published between 2007 and 2020 were eligible for our review: seven studies [8, 11–16] reported LH, and seven studies [9, 17–22] reported HeLP (Fig. 1). There were 10 cohorts (seven prospective [11, 12, 17–21], two retrospective [8, 9], one not specified [13]), three randomized controlled trials (RCT) comparing LH with HC [14–16] and MP [16], and one RCT comparing HeLP with RBL [22]. Of the 1540 patients included in these studies, majority were classified as suffering from grades II and III HD (35.8% and 33.6%, respectively), while grades I and IV HD patients were less frequent (5.1% and 0.4%, respectively, grade unavailable for 25.1% of cases). The studies’ characteristics and patient demographic details are depicted in Table 1.

**Table 1:** Characteristics of included studies

Author	Year of publication	Journal of publication	Country	Study design	Mono-/multicentric	Study period	Sample Size (*n*)	Age (mean in years ± SD)	Sex (*n*)		Hemorrhoidal grade			
									Male	Female	I	II	III	IV
Laser hemorrhoidoplasty														
Karahaliloglu et al. ^8^	2007	Coloproctology	Turkey	ORC	Monocentric	2005	106	20–80^a^	-	-	74	32	0	0
Jahanshahi et al. ^11^	2012	Polish Journal of Surgery	Iran	OPC	Monocentric	2010–2011	341	-	219	122	0	127^b^	94^b^	2^b^
Brusciano et al. ^12^	2019	Uptades in Surgery	Italy	OPC	Monocentric	2018	50	42 ± 12.6	28	22	0	5	45	0
Plapler et al. ^13^	2009	Photomedicine and Laser Surgery	Brazil	-	Monocentric	-	15 LH10 open HC	25–45^a^ -	-	-	0	-	-	0
Naderan et al. ^14^	2016	Journal of Investigative Surgery	Iran	RCT	Monocentric	2011–2012	30 LH30 open HC	43.7 ± 13.744.3 ± 11.3	1311	1719	0	1310	1720	0
Alsisy et al. ^15^	2018	Menoufia Medical Journal	Egypt	RCT	Monocentric	2016–2017	30 LH30 open HC	34.7 ± 10.233.7 ± 10.2	1815	1215	0	13 17	17 13	0
Poskus et al. ^16^	2020	International Journal of Colorectal Disease	Lithuania	RCT	Monocentric	2016–2018	40 LH 41 MP 40 HC	47 ± 13 49 ± 13 45 ± 12	27 22 21	13 19 19	0	10 10 7	30 31 33	0
Hemorrhoidal laser procedure														
Salfi et al. ^9^	2009	Coloproctology	Italy	ORC	Monocentric	2005–2006	200	21–81^a^	72	128	0	-	-	0
Giamundo et al. ^17^	2011	Surgical Endoscopy	Italy	OPC	Monocentric	2009–2010	30	47 ± 12.6	16	14	0	14	16^c^	0
Crea et al. ^18^	2014	The American Journal of Surgery	Italy	OPC	Monocentric	2010–2012	97	47 (28–74)^d^	53	44	0	51	46^c^	0
De Nardi et al. ^19^	2016	Techniques in Coloproctology	Italy	OPC	Multicentric	2012–2014	51	44^d^ (18–70)^d^	36	15	0	29	22^c^	0
Boarini et al. ^20^	2017	Journal of Inflammatory Bowel Diseases & Disorders	Brazil	OPC	Monocentric	2011–2014	55	45.5 (22–67)^d^	27	28	44		11^c^	0
Giamundo et al. ^21^	2018	Techniques in coloproctology	Italy	OPC	Multicentric	2016–1017	284	47.5 (17–77)^d^	183	101	5	174	101^c^	4^c^
Giamundo et al. ^22^	2011	Disease of the colon and rectum	Italy	RCT	Multicentric	2009–2010	30 HeLP 30 RBL	47^d^ 44^d^	13 12	17 18	0	20 19	10^c^ 11^c^	0

*SD* standard deviation, *ORC* observational retrospective cohort, “-” not available, *OPC* observational prospective cohort, *LH* laser hemorrhoidoplasty, *HC* hemorrhoidectomy, *RCT* randomized controlled trial, *MP* mucopexy, *HeLP* hemorrhoidal laser procedure, *RBL* rubber band ligation

^a^Expressed as range

^b^118 additional mixed grades of hemorrhoids were included

^c^Severe prolapses were excluded

^d^Expressed as median (with range)

### Perioperative characteristics

#### Laser hemorrhoidoplasty

LH was performed under general anesthesia [11, 14, 16], spinal anesthesia [11, 15], local anesthesia with [12, 13] or without [8] sedation, or without anesthesia [8] (Table 2). Antibioprophylaxis was administered in three studies [12, 14, 16]. The operation time was reported in three observational cohorts [8, 11, 12] and three RCTs [14–16], which ranged from 5 [11] to 40.4 min [14]. Moreover, the three RCTs [14–16] showed a significantly shorter operation time with LH versus open HC (mean 33.1 min ± 7.3 versus mean 52.6 min ± 15.6, *p* < 0.001 [14]; mean 30.6 min ± 4.9 versus mean 50.5 min ± 12.1, *p* < 0.001 [15]; mean 15 min ± 5.6 versus mean 29 min ± 10.3, *p* < 0.001 [16], respectively). Four studies [11–13, 15] described the hospitalization duration, which ranged from 3 [13] to 48 h [12] and was similar to open HC [15].

**Table 2:** Perioperative characteristics of laser hemorrhoidoplasty

Study	Preoperative enema	Antibioprophylaxis (molecule)	Anesthesia (% with topical, local, spinal, sedation, general, or without anesthesia)	Laser technique		Number of hemorrhoidal columns (%)			Operation time (mean in minutes ± SD)	Hospitalization duration (mean in hours ± SD)
				Wavelength (nm), power (W), duration (s), pause (s)	Number of shots per hemorrhoidal node	I	II	III		
Karahaliloglu et al. ^8^	-	-	81.1% local (Prilocain), 18.9% without	980, 15, 3, -	≥ 6^a^	-			6.8	-
Jahanshahi et al. ^11^	-	-	100% general or spinal	980, 15, 3, -	1^a^	-			10 (5–15)^b^	18
Brusciano et al. ^12^	-	Yes (Ceftriaxone)	100% local (Ropivacaine) + sedation (Propofol)	1470, 8, 3, -	10–12	0	16%	84%	14	48
Plapler et al. ^13^	-	No	LH: 100% local (Xylocaine) + sedation (Propofol) Open HC: -	810, 5, -, -	-	0 -	40% -	60%^c^ -	-	3
Naderan et al. ^14^	-	Yes (Ceftriaxone + Metroniadzole)	LH: 100% general Open HC: 100% general	980, 15, 1.2, 0.6	3^a^	25% 20%	75% 80%	0	33.1 ± 7.3 52.6 ± 15.6^d^	-
Alsisy et al. ^15^	-	-	LH: 100% spinal Open HC: 100% spinal	980, 15, 1.2, 0.6	3^a^	50% 40%	40% 43.3%	10% 16.7%	30.6 ± 4.9 50.5 ± 12.1^d^	26.4 ± 7.2 28.8 ± 9.6
Poskus et al. ^16^	No	Yes (Cefazolin + Gentamycin + Metronidazole)	LH: 100% general MP: 100% general Open HC: 100% general	1470, 8, 3, 1	-	-			15 ± 5.6 16 ± 5.6 29 ± 10.3^d^	-

*nm* nanometer, *W* watt, *s* second, *SD* standard deviation, “-” not available, *LH* laser hemorrhoidoplasty, *HC* hemorrhoidectomy, *MP* mucopexy

^a^Additional shots were given in case of larger hemorrhoids

^b^Expressed as range

^c^One circular prolapse was classified as III columns

^d^The difference was statistically significant

#### Hemorrhoidal laser procedure

HeLP was performed under topical anesthesia [19, 21], local anesthesia [21], spinal anesthesia [21], sedation [19–21], or without anesthesia [9, 17, 18, 20, 22] (Table 3). Four studies prescribed preoperative enema [18, 19, 21, 22] and/or antibioprophylaxis [18–21]. The operation time ranged from 7 [20, 21] to 40 min [18] and was similar to RBL [22] (HeLP: median 10 min, range 7.8–11.2 versus RBL: median 8 min, range 5.8–9.6, *p* = 0.96). Hospitalization duration was up to 24 h [17–20], but was not described in three studies [9, 21, 22].

**Table 3:** Perioperative characteristics of hemorrhoidal laser procedure

Study	Preoperative enema	Antibioprophylaxis (molecule)	Anesthesia (% with topical, local, spinal, analgesia, sedation, general, or without anesthesia)	Laser technique			Operation time (mean in minutes ± SD)	Hospitalization duration (mean in hours)
				Wavelength (nm), power (W), duration (s), pause (s)	Number of shots per hemorrhoidal node	Number of vessels treated (mean ± SD)		
Salfi et al. ^9^	-	-	100% without	980, 10–25, -, -	4	-	-	-^a^
Giamundo et al. ^17^	-	No	90% without, 10% analgesia (Ketorolak and/or Paracetamol)	980, 13, 1.2, 0.6	5 + 2^b^	10.8 ± 1.2	9.5 ± 2.3	2-24^c^
Crea et al. ^18^	Yes	Yes	88.7% without, 11.3% analgesia (Ketorolac)	980, 13, 1.2, 6	5 + 2^b^	10 (5–13)^d^	18 (12–40)^d^	6-24^c^
De Nardi et al. ^19^	Yes	Yes (Metronidazole)	100% topical (Lidocaine/Prilocarpine), 37.3% sedation (Midazolam)	980, 13, 1.2, 0.6	5 + 3^b^	13 (10–15)^d^	21.29 ± 5.6	0-6^c^
Boarini et al. ^20^	-	Yes (Cefazolin)	96.4% without, 3.6% sedation (Midazolam, Fentanyl)	980, 13, 1.2, 0.6	5 + 1^b^	10.1 (7–12)^c^	9.9 (7–19)^c^	4
Giamundo et al. ^21^	Yes	Yes (26.4%) No (73.6%)	86.6% topical (Lidocaine/Prilocarpine), 1.4% spinal or local, 12.0%: sedation (Midazolam)	980, 13, 1.2, 0.6	5 + 3^b^	12	15.5 (7–31)^d^	-
Giamundo et al. ^22^	Yes	-	HeLP: 100% without RBL: 100% without	980, 13, 1.2 0.6	5	-	10 (7.8–11.2)^d^ 8 (5.8–9.6)^d^	-^a^

*nm* nanometer, *W* watt, *s* second, *SD* standard deviation, “-” not available, *HeLP* hemorrhoidal laser procedure, *RBL* rubber band ligation

^a^Duration of the hospitalization not mentioned, but performed as outpatient procedure

^b^Additional shots were given in case of persisting Doppler signal

^c^Expressed as range

^d^expressed as median (with range)

### Postoperative pain and return to normal activities

Postoperative pain was significantly lower after LH compared with HC [13, 15, 16] and resulted in a shorter return to normal activities [15, 16]. Early postoperative visual analog score (VAS) was also decreased with HeLP compared with RBL [22] (mean 1.1 versus 2.9, *p* < 0.001, respectively). Details of postoperative pain and return to normal activities are depicted in Table 4.

**Table 4:** Postoperative pain and return to normal activities

Study		Pain		Postoperative analgesia	Return to normal activities (mean in days ± SD)
		Score (mean VAS scale from 0 to 10 ± SD)	Timepoint		
Laser hemorrhoidoplasty					
Karahaliloglu et al. ^8^		-		-	-
Jahanshahi et al. ^11^		-		-	-
Brusciano et al. ^12^		2 (0–3)^a^ 0	0–3 days 4 days	-	1-2^a^
Plapler et al. ^13^	LH/HC	0.8 ± 1.1/1.8 ± 0.7^c^	0–28 days	Diclofenac^b^	-
Naderan et al. ^14^	LH/HC	5.7 ± 1.5/5.2 ± 1.3 4.2 ± 1.4/5.2 ± 1.2 2.9 ± 1.5/4.1 ± 1.3 1.6 ± 1.5/2.7 ± 1.5	6 h 12 h 18 h 24 h	Morphine (in the recovery room)^b^	-
Alsisy et al. ^15^	LH/HC	2 (1–8)^a^/6 (3–10)^a, c^	1 day	Oral analgesia^b^	7.5 ± 1.8 vs 22.9 ± 3.9^c^
Poskus et al. ^16^	LH/MP/HC	3.1/2.7/5.0^c^	-	-	15 (5–14)^a^ vs 24 (9–30)^a^ vs 30 (14–35)^a, c^
Hemorrhoidal laser procedure					
Salfi et al. ^9^		-		-	-
Giamundo et al. ^17^		1.4 ± 1.7	0–3 days	-	-
Crea et al. ^18^		0 (0–2)^d, e^ 0 (0–2)^d, e^	1 week 1 month	-	Immediately
De Nardi et al. ^19^		0.1 (0–1)^a, e^ 0.1 (0–2)^a, e^0.0^e^	3 months 12 months 24 months	Paracetamol^b^	0–3^a^
Boarini et al. ^20^		1.4 (0–3)^a^	0–2 h	-	-
Giamundo et al. ^21^		1.1 (0–5)^d^ 0.9 ± 0.8 1.1 ± 1.0	2 weeks 6 months 12 months	Paracetamol^b^	-
Giamundo et al. ^22^	HeLP/RBL	1.1 (0–2)^d^/2.9 (1–5)^c, d^ 0.8 (0–2)^d^/1.0 (0–3)^d^	1–3 days 2 weeks	-	-

*VAS* visual analog scale, *SD* standard deviation, “-” not available, *LH* laser hemorrhoidoplasty, *HC* hemorrhoidectomy, *MP* mucopexy, *HeLP* hemorrhoidal laser procedure, *RBL* rubber band ligation

^a^Expressed as range

^b^Administered on request

^c^the difference was statistically significant

^d^Expressed as median (range)

^e^VAS scale ranged from 0 to 3

### Intraoperative and postoperative complications

#### Intraoperative complications

Bleeding was the only intraoperative complication reported. It was reported by four studies [8, 12, 14, 15] describing LH and for all studies [9, 17–22] describing HeLP (Table 5). Its incidence ranged from 0 [12] to 1.9% [8] and from 5.5 [20] to 16.7% [22], respectively. Two RCTs [14, 15] reported a significant lower blood loss volume during LH versus HC (mean 12.4 ml ± 4.5 versus 22.8 ml ± 8.3, *p* < 0.001 [14]; mean 15.5 ml ± 4.8 versus mean 36.5 ml ± 7.2, *p* < 0.001 [15], respectively). However, in the RCT by Giamundo et al. [22], the intraoperative blood loss was similar for HeLP versus RBL (16.7% versus 10%, *p* = 0.12, respectively).

**Table 5:** Intraoperative and postoperative complications of laser procedures

Study		% of intraoperative complications (treatment for the complication if reported)	% of postoperative complications (treatment for the complication if reported)						Follow-up (in months)
		Bleeding	Bleeding	Hemorrhoidal thrombosis	Infection	Urinary retention	Other	Overall	
Laser hemorrhoidoplasty									
Karahaliloglu et al. ^8^		1.9% (Ø)	0	0	0	0	Mucosal damage: 0.9% (ligature)	0.9%	12
Jahanshahi et al. ^11^		-	0.6% (suture)	0	0.6%	0	Edema: 2.3%	3.5%	12
Brusciano et al. ^12^		0	64% (Ø)^b^	0	0	0		64%	8.6^a^
Plapler et al. ^13^	LH HC	-	0	0	0	0	Burn lesion: 26.7% Plicoma/skin tag: 33.3% 0	60% 0%	1
Naderan et al. ^14^	LH HC	12.8 ± 4.5 22.8 ± 8.3^c, d^	10% (cons.) 30% (cons.)	6.7% (cons.) 0%	0	3.3% 10%		20% 40%	12
Alsisy et al. ^15^	LH HC	15.5 ± 4.8 36.5 ± 7.2^c, d^	0 3.3% (packing and hemostatic drug)	10% (cons.) 0	0 10% (antibiotherapy)	0 13.3%^d^	0 Anal stenosis: 13.3% (lateral sphincterotomy) ^d^	10% 40%	3
Poskus et al. ^16^		-	0	0	0	0		0%	12
Hemorrhoidal laser procedure									
Salfi et al. ^9^		8.5% (1% ligation, 7.5% laser)	0.5%^e^	0	0	0		0.5%	12
Giamundo et al. ^17^		13.4% (6.7% laser, 6.7% suture)	0	0	0	0		0%	5.8 (1–12)^a^
Crea et al. ^18^		12.4% (9.3% laser, 3.1% suture)	0	0	0	0		0%	15 (6–30)^∏^
De Nardi et al. ^19^		5.9% (2% laser, 3.9% suture)	7.8%	7.8%	0	0		15.6%	26.3 ± 9.1^a^
Boarini et al. ^20^		5.5% (laser)	20% (Ø)	0	0	0		20%	6
Giamundo et al. ^21^		8.8% (2.5% Ø or laser, 6.3% ligature or suture)	3.5% (flavonoid)	1.4%	0	0	Anismus: 1.4% Sensation of incomplete evacuation: 3.1% (Ø)	9.4%	12
Giamundo et al. ^22^	HeLP RBL	16.7%: (10% surgical, 6.7% laser) 10% (3.3% surgical)	23.3% (Ø) 26.7% (Ø)	0	0	0		23.3% 26.7%	6

“-” not available, *cons.* conservative, Ø = no treatment

^a^Expressed as mean ± SD or (range)

^b^Post-defecatory bleeding only, and stopped spontaneously from the 7th postoperative day, 0% of spontaneous bleeding

^c^Expressed in volume (mean milliliters ± standard deviation)

^d^The difference was statistically significant

^e^Additional 11.0% of discharge was described. They were not considered as complications since it did not cause discomfort, and it disappeared within 30 days. Moreover, it was not specified whether the discharge consisted of blood or mucous

#### Postoperative complications

All studies reported postoperative complications (Table 5). The incidence of complications ranged between 0 [16] and 64% [11] after LH and between 0 [17, 18] and 23.3% [22] after HeLP. The most common reported complication was bleeding (range 0–64% after LH and 0–23.3% after HeLP), which resolved with suture [11], packing and/or haemostatic drug [15, 21], conservative therapy [14], or without treatment [12, 20, 22]. Further complications were thrombosis (range 6.7–10% after LH [14, 15], range 1.4–7.8% after HeLP [19, 21]), infection (up to 0.6% after LH [11], not reported after HeLP), and urinary retention (up to 3.3% after LH [14], not reported after HeLP). The latter was significantly increased after HC versus LH (13.3% versus 0%, *p* = 0.038, respectively) [15]. Other complications reported after LH included mucosal damage (0.9%, treated with ligature) [8], edema (2.3%) [11], burn lesion (26.7%) [13], and skin tag (33.3%) [13]. Other complications reported after HeLP included anismus (1.4%) [21] and sensation of incomplete evacuation (3.1%, which did not require any treatment) [21].

### Surgical indicators of postoperative outcomes

Surgical indicators of postoperative outcomes are detailed in Table 6. The postoperative follow-up duration ranged between 1 [13] and 12 months [8, 11, 14, 16] after LH and between 1 [17] and a mean of 35.4 months [19] after HeLP.

**Table 6:** Surgical indicators of postoperative outcomes: improvement, persistence, resolution, recurrence, and reoperation

Study		HD downgrading	Symptoms improvement	Persistence	Resolution	Recurrence	Reoperation (timepoint; type)	Follow-up (in months)
Laser hemorrhoidoplasty								
Karahaliloglu et al. ^8^		-	-	-	-	11.3%^a^	54.7% (within 3 months, LH)	12
Jahanshahi et al. ^11^		-	-	-	-	0%	-	12
Brusciano et al. ^12^		-	-	-	-	0%	-	8.6^b^
Plapler et al. ^13^	LH HC	-	-	-	60.4%^c^ -	-	-	1
Naderan et al. ^14^	LH HC	-	-	-	70%^a^ 76.7%^a^	-	0% 0%	12
Alsisy et al. ^15^	LH HC	-	-	0%^a^ 10%^a^	100%^a^ 90%^a^	0% 0%	0% 0%	3
Poskus et al. ^16^	LH MP HC	-	-	-	72.5%^a^ 58.5%^a^ 82.5%^a^	10%^a^ 22%^a^ 0%^a, d^	-	12
Hemorrhoidal laser procedure								
Salfi et al. ^9^		-	91%	-	-	9.4%	-	12
Giamundo et al. ^17^		77%	91.7%	7%^a, c^	-	8.3%^a^	-	5.8 (1–12)^b^
Crea et al. ^18^		>85%	85%	-	> 90%^a^	5%	-	15 (6–30)^e^
De Nardi et al. ^19^		-	86.3%	9.8%^a^	76.9%^c^	7.8%	7.8% (2 [1–5]^b^ months; 2% RBL, 3.8% THD, 2% HC)	26.3 ± 9.1^b^
Boarini et al. ^20^		80%	-	-	83.6%^a^	-	-	6
Giamundo et al. ^21^		-	-	9.7%^a, c, e^	90.3%^a^	-	2.8% (6^b^ months; 0.7% HeLP, 0.7% SH, 0.7% THD, 0.7% HC)	12
Giamundo et al. ^22^	HeLP RBL	80% 40%^d^	-	-	90.0%^a^ 53.3%^a, d^	-	-	6

*HD* hemorrhoidal disease, “-” not available, *LH* laser hemorrhoidoplasty, *HC* hemorrhoidectomy, *MP* mucopexy, *RBL* rubber band ligation, *THD* transanal hemorrhoidal dearterialization, *HeLP* hemorrhoidal laser procedure, *SH* stapled hemorrhoidopexy

^a^Symptomatic recurrence, resolution, or persistence

^b^Expressed as mean ± standard deviation or (range)

^c^Prolapse recurrence, resolution, or persistence

^d^The difference was statistically significant

^e^Expressed as median (range)

^e^Described as persistence in the results section, but as persistence or recurrence in the discussion section

#### HD downgrading

HD downgrading for at least one grade derived from the Goligher classification was reported by four studies after HeLP [17, 18, 20, 22]. It was 80% at 6 months [20, 22], 77% at a mean of 5.8 months [17], and > 85% at 15 months [18]. In the RCT by Giamundo et al. [22], HD grade was significantly decreased after HeLP compared with RBL (80% versus 40% at 6 months, *p* < 0.001, respectively). No study reported HD downgrading after LH.

#### Symptom improvement

Improvement of HD symptoms was reported by four studies [9, 17–19] after HeLP and ranged from 85% at a median of 15 months [18] to 91.7% at a mean of 5.8 months [17]. No study reported symptom improvement after LH.

#### Persistence

Symptomatic ± prolapse persistence was 0% at 3 months [15] and 7% of at a mean of 5.8 months [17] after LH. After HeLP, two studies [19, 21] reported 10% of recurrences up to 26 postoperative months.

#### Resolution

Resolution of hemorrhoidal prolapse was reported in 60.4% of patients at 1 month after LH [13] and 76.9% of patients at a mean of 26.3 months after HeLP [21]. After LH, resolution of symptoms was 100% at 3 months [15] and ranged between 70 [14] and 72.5% [16] at 12 months. The latter results were similar compared with HC or MP [14–16]. In the other hand, resolution of symptoms after HeLP was statistically higher after HeLP versus RBL at 6 months (90% versus 53.3%, *p* < 0.001, respectively) [22]. Overall, symptomatic resolution ranged between 83.6% at 6 months [20] and 90.3% at 12 months [21]. Nevertheless, resolution rate was unavailable in three studies after LH [8, 11, 12] and in two studies after HeLP [9, 17].

#### Recurrence

After LH, two studies reported symptomatic recurrences, which ranged between 10 [16] and 11.3% [8] at 12 months. In the RCT by Poskus et al. [16], recurrence rates were significantly lower after HC, compared with LH and MP (0% versus 10% versus 22%, *p* < 0.004, respectively). No recurrence was reported after LH for three studies [11, 12, 15]. However, data on recurrence were unavailable for two studies [13, 14].

After HeLP, one study [17] reported a symptomatic recurrence rate of 8.3% at a mean of 5.8 months, and three other studies [9, 18, 19] reported recurrences from 5% at a median of 15 months to 9.4% at 12 months. Data on recurrence were unavailable for three studies [20–22].

#### Reoperation

Data on reoperation were available in three studies after LH [8, 14, 15] and in two studies after HeLP [19, 21]. In the study by Karahaliloglu et al. [8], 54.7% of patients were reoperated with redo LH, for insufficient treated nodes between 1 and 3 months. Two other studies [14, 15] reported no need for reoperation after LH, with a follow-up of 3 to 12 months. After HeLP, up to 7.8% of cases were reoperated within 5 months [19], with redo HeLP, RBL, transanal hemorrhoidal dearterialization, stapled hemorrhoidopexy, or HC [19, 21].

## Discussion

The present systematic review included 14 studies describing LH [8, 11–16] and HeLP [9, 17–22], representing 1570 patients with grades I to IV HD. Primarily, laser therapies seemed to be safe. The single intraoperative complication was bleeding (0–1.9% for LH, 5.5–16.7% for HeLP), managed with intraoperative laser, suture, or conservatively. Postoperative complications occurred up to 64% of patients after LH and up to 23.3% after HeLP. However, these complications were frequently minor, which did not require therapy or were treated with conservative measures. Only 0.9% of postoperative complications required surgical therapy [8]. Moreover, 64% of bleeding reported after LH [12] consisted of post-defecatory bleeding, which did not require treatment and spontaneously resolved after the 7th postoperative day. Compared with other non-excisional therapy for HD, the RCT by Giamundo et al. [22] showed similar intraoperative bleeding rate associated with HeLP versus RBL (16.6% versus 10%, *p* = 0.12). Compared with excisional therapy, two RCTs [14, 15] showed lower intraoperative blood volume loss associated with LH versus HC. Moreover, postoperative complications were decreased with LH (urinary retention: 0% for LH versus 13.3% for HC, *p* = 0.038; anal stenosis: 0% for LH versus 13.3% for HC, *p* = 0.038) [15].

Secondarily, laser therapies are effective for the treatment of grades II and III HD, as shown by surgical indicators of postoperative outcomes. Resolution of symptoms ranged between 70 and 100% after LH and from 83.6 to 90% after HeLP. These rates are similar to the success rate of RBL reported in the literature. As shown by a retrospective study [23] of 750 patients with grades I to III HD treated with RBL, the success rate was 89%. Moreover, four RCTs included in our review reported similar resolution after LH compared with HC [14–16] or MP [16] and after HeLP compared with RBL [22]. Another surgical indicator was the recurrence rate, reported between 0 and 11.3% after LH and between 5 and 9.4% after HeLP. In the literature, recurrence rate at 12 months was reported up to 5% after mucopexy [24] and up to 11.1% [25] after hemorrhoidal artery ligation ± mucopexy. Compared with HC, LH showed conflicting results with one RCT reporting similar recurrence rate [15], but another RCT reporting decreased recurrences associated with HC [16]. Another surgical indicator of postoperative outcome was the reoperation rate, reported in 54% of patients after LH [8]. However, Karahaliloglu et al. [8] were the first to report their experience with LH, and this high reoperation rate seemed to decrease with progress in the learning curve. Moreover, two other studies reported no need for reoperation after LH, at 3 and 6 months of follow-up [14, 15]. Nevertheless, a long-term follow-up is mandatory to identify recurrences and the potential need for further intervention.

Thirdly, laser therapies conferred the advantages of a quick return to normal activities and low postoperative pain. The latter is explained by the absence of excision of tissue below the dentate line, where pain fibers are present [26]. Compared with HC, two RCTs showed decreased postoperative pain score associated with LH [15, 16]. Compared with RBL, another RCT showed decreased postoperative pain associated with HeLP [22]. However, pain comparison between studies is hazardous as postoperative analgesia varied significantly among studies.

The main limitation of the study is the heterogeneity of included studies. Perioperative characteristics, such as preoperative enema, antibioprophylaxis, anesthesia, and laser techniques, varied significantly among studies. Moreover, while grades II and III HD are good candidates for laser therapies, some studies included grades I and IV HD [8, 11, 20, 21]. Another weakness is the small population size of included studies, with the largest cohort composed of 341 patients [11]. This resulted in a decreased statistical power. Moreover, rare complications may be unidentified.

In this review, surgical indicators were used as surrogates of postoperative outcomes. Nevertheless, they were irregularly reported among studies. Moreover, Giamundo et al. [21] reported 9.7% of symptomatic persistence in the results section, but this was latter mentioned as persistence and/or recurrence in the discussion section. Inconsistency with outcomes definition precluded a meticulous analysis. As demonstrated by a recent systematic review [27], assessment of treatment efficiency should emphasize the use of validated scoring systems. However, none of the included study used these scores.

Overall, laser therapies appeared to be safe and effective techniques for the treatment of HD. Moreover, the learning curve is quick and was estimated from three to five cases [14]. These techniques could be alternatives to RBL or hemorrhoidal artery ligation ± mucopexy for the treatment of grade II or III HD. Only one RCT compared HeLP with RBL [21] and future research should focus on the comparison between laser and other non-excisional therapies of HD. Another unanswered question is the utility of the Doppler for the laser procedure. As reported by two RCTs [28, 29], the Doppler use did not show benefits for the hemorrhoidal artery ligation technique. Finally, benefits of LH or HeLP should be compared.

## Conclusions

To conclude, non-excisional laser therapies, including LH and HeLP, are safe and effective. They should be considered for the treatment of grades II and III HD unresponsive to conservative management.

## Electronic supplementary material

ESM 1 (DOC 64 kb)

ESM 2 (DOCX 71 kb)
