# Supplementary material for: Non-excisional laser therapies for hemorrhoidal disease: a systematic review of the literature
Source: Lasers Med Sci. 2020 Sep 10;36(3):485–96. doi: 10.1007/s10103-020-03142-8 (PMC7952353; doi:10.1007/s10103-020-03142-8)
Supplement: Supplementary file 2 — (DOCX 71 kb) [file 10103_2020_3142_MOESM2_ESM.docx]

Supplementary Table 2.

| General informations of the study | First author  Year of publication  Journal of publication  Country |
| --- | --- |
| Methodological informations | Study design  Mono-/multicentric  Study period  Sample size |
| Baseline characteristics of the study population | Age  Gender  Hemorrhoidal grade adapted from the Goligher classification ^3^ |
| *Primary endpoints* | |
| Surgical indicators of postoperative outcomes | HD downgrading  Symptoms improvement  Persistence  Resolution  Recurrence  Reoperation  Follow-up duration |
| *Secondary endpoints* | |
| Perioperative characteristics | Preoperative enema  Antibioprophylaxis  Type of anaesthesia  Laser technique: wavelength, power, duration and pause; number of shots per hemorrhoidal node; number of vessels treated  Number of hemorrhoidal columns  Operative time  Hospitalization duration |
| Complications | Intraoperative complications  Postoperative complications: bleeding, hemorrhoidal thrombosis, infection, urinary retention, other |
| Postoperative pain and return to activities | Pain score and timepoint  Postoperative analgesia  Return to normal activities |
